# Shared leadership and project success: The mediational role of individual- and team-related factors

**DOI:** 10.1371/journal.pone.0342268

**Published:** 2026-02-13

**Authors:** Beata Bajcar, Jolanta Babiak, Agata Klaus-Rosińska, Joanna Iwko, Dorota Kuchta

**Affiliations:** Faculty of Management, Wrocław University of Science and Technology, Poland; STIKES Wira Medika PPNI Bali: Sekolah Tinggi Ilmu Kesehatan Wira Medika PPNI Bali, INDONESIA

## Abstract

The existing research on the role of shared leadership in achieving project success is rudimentary but suggests that this role may be important. In this study, we examined the relationship between shared leadership and project success, considering four potential mediators: individual engagement, justice (understood as perceived fairness), team building, and teamwork. We conceptualized project success as a construct with four dimensions: project performance, stakeholder satisfaction, project outcomes, and the subjective evaluation of project success. Alternatively, we allow it to refer to any one of these dimensions separately at the discretion of the decision maker. Our study employed structural equation modelling, surveys of 320 team members in Poland, and established measurement tools for the model variables. Our findings revealed simple mediating effects of individual engagement, team building, and teamwork in the relationship between shared leadership and project success. Additionally, this relationship was serially mediated by chains of factors: individual engagement and team building; justice and team building; individual engagement and teamwork; and justice and teamwork. We identified several strategies for various definitions of project success, which ensure efficient project management when shared leadership is applied. We also discuss the implications of our findings for project management theory and practice.

## Introduction

Project success is the goal of every project manager, project team, organisation, and stakeholder [1]. Factors contributing to project success, known as project success factors, have been extensively researched. Scientists have identified numerous project success factors and have attempted to measure their respective impacts on project success. Various efforts to categorize these factors have been undertaken. In nearly all classifications of project success factors, whether based on the percentage of project managers naming a factor [2], the number of publications mentioning it [1], or its influence on project success [2], the most significant factors are linked to the interplay between project management, leadership, the project team, and individual team members.

Leadership is considered one of the most essential skills for effective project management and achieving project success [3–9]. Various types of leadership have been considered in their role as project success factors. Recent management reports [10,11] suggest that shared leadership, when applied under appropriate conditions, can be beneficial for organisations. It is seen as a management style that enhances organisational performance, fosters innovation and adaptability, promotes trust and collaboration, and improves job satisfaction and motivation. Despite these potential benefits, surprisingly little scientific research has been conducted on its role as a project success factor. Therefore, the aim of this study is to extend existing research by investigating the relationship between shared leadership and project success, and to analyse the mediating role of selected factors (project engagement, justice, understood as perceived fairness, team building, and teamwork) in this relationship.

In recent literature on project success factors, project success is consistently presented, in line with current perspectives [12], as a multicriteria construct based on several criteria. We follow this general trend by evaluating project success through an aggregation of assessments of project performance, stakeholder satisfaction, project outcomes, and subjective success. We added the possibility of considering either the equally weighted aggregation of the criteria or a single criterion. This approach reflects the reality that each project stakeholder may have a different understanding of project success [13].

**Fig 1:**
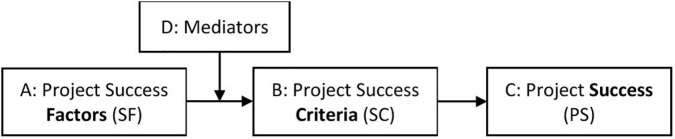
General model of project success (own elaboration based on [14]).

According to Lim and Zain Mohamed [14], we can place our reasoning in the broader context of the following model (Fig 1).

In box A of Fig 1 project success factors are presented, which are defined by Lim and Zain Mohamed [14] according to the Concise English Dictionary [15] as “any circumstance, fact, or influence that contributes to project success.” Box B contains project success criteria that are described as “the set of principles or standards by which project success is or can be judged” [15]. Project success, depicted in box C, is determined by each project stakeholder based on box B, using various weights (ranging from 0 to 1) assigned to individual project success criteria. Some weights may be 0, effectively omitting the respective criterion from the project success evaluation. The total of the weights should equal 1.

To determine which project success factors (box A) contribute to project success (box C), and to what extent, we first need to define project success criteria (box B) [16]. Next, it is necessary to investigate the influence of project success factors (box A) on individual project success criteria (box B), and finally on project success (box C). Thus, we have reformulated the definition of project success factors as “any circumstance, fact, or influence which contributes to the fulfilment of the accepted project success criteria or their predefined aggregation.” The influence of project success factors on project success criteria or their aggregation may be mediated by several variables, called mediators (box D), defined as “variables that intervene between the independent variable(s) and the dependent variable(s) to help explain the relationship between them” [16]. In our case, the independent variables are project success factors, which may affect the mediators, which in turn may affect project success criteria and, consequently, their aggregation (the dependent variables). It should be noted that the model in Fig 1 may also include moderators [17], which is, however, outside the scope of this paper.

In our study, we investigate, practically for the first time in the literature on quantitative analysis of project success factors, the role of selected success factors in achieving both an aggregation of the selected project success criteria (with equal weights) and each criterion individually.

Having outlined the problem of the relationship between box B and box C (Fig 1), we will now discuss the implementation of boxes A, B, and D from the model in Fig 1 into our study.

### Project success criteria

Box B has undergone a long and vivid evolution since the time when project success was understood in a limited, iron-triangle-based (time, cost, scope) manner. Ika and Pinto [12] culminate the development of project success notion and criteria with their own proposal, advising the use of four categories of project success criteria (Box B):

i those referring to ‘project plan success’: essentially, here we find criteria based on the iron triangle idea, linked to the fulfilment of project plan;
ii those referring to the satisfaction of project stakeholders;
iii those referring to business case success, which is linked to the evaluation by key project stakeholders of project outcomes, understood as ‘broader effects or changes, strategic and long-term in nature, that occur as a result of implementing project’s deliverables’;
iv those referring to the evaluation of project success by the society, or generally to project green efficacy.

We decided to consider four categories of project success criteria: three corresponding to the first three categories listed above (green management, the fourth category, is still not widely applied in organisations worldwide [18]), and one additional criterion: the subjective evaluation of project success by the respondent. This latter criterion was selected in line with Lim & Zain Mohamed [14] and Serra & Kunc [19], acknowledging that project success is a complex and strongly context-dependent construct, requiring subjective evaluations by those involved in the project.

The elements of the first three criteria categories were selected for our model on the basis of the literature on project success [14,19–30]: the selection criteria were based on the frequency with which a factor was mentioned or the authors’ opinions on the importance of the factor. Thus, the criteria categories were concretized as follows:

i *Project performance*: adhering to the planned deadline, planned cost, planned quality, and planned scope, as well as achieving the planned effect (adequacy of the project deliverables against the reason for starting the project and the shortness of the period needed for the project deliverables acceptance);
ii *Stakeholders’ satisfaction*: satisfaction of the customer/end users and key sponsors with the project deliverables, and satisfaction of project team members and the project manager with both the process of project implementation and the project deliverables;
iii *Project outcomes*: the actual use of the project deliverables by the intended users, a justified conviction that the project deliverables will be useful for a longer period, and the belief that the project has better prepared the organisation for future operations.
iv *Success evaluation*: subjective evaluation of project success.

More details on the method of measuring project success will be given in the “Methods” section. Let us now proceed to justify our choice of shared leadership as a potential project success factor (Box A) and provide a brief description of this leadership style.

### Shared leadership and project success

Shared leadership began to emerge in leadership theories in the mid-1990s and has been defined in various ways (see [31] for a review). Here we adopt the following definition: “shared leadership occurs when group members actively and intentionally shift the role of leader to one another as necessitated by the environment or circumstances in which the group operates” [32]. Other leadership styles overlap to some extent with shared leadership [31] and are even considered by some authors to be its components [33,34], such as collective leadership, emergent leadership, self-leadership, empowering leadership, and participative leadership. Various leadership styles are considered in the literature as potential factors contributing to project success (see Table 1).

**Table 1:** Relevant studies on the relationship between leadership styles and project success.

Leadership style	Studies
Transformational	Abbas & Ali, 2021 [64]; Aga et al., 2016 [51]; Al Shanqaiti & Farea, 2021 [68]; Doan et al., 2020 [69]; Fareed et al., 2021 [70]; Fareed & Su, 2022 [71]; Khan et al., 2015 [72]; Maqbool et al., 2017 [73]; Nauman et al., 2022 [4]; Rogo et al., 2020 [74]; Zaman et al., 2019 [75]; Zavari & Afshar, 2021 [76]; Zhao et al., 2021 [77]
Transactional	Abbas & Ali, 2021 [64]; Aga, 2016 [26]; Liphadzi et al., 2015 [78]; Mufaricha et al., 2021 [79]
Inclusive	Khan et al., 2020 [80]; Mir et al., 2021 [81]; Rehman, 2020 [82]
Entrepreneurial	Latif et al., 2020 [83]; Latif et al., 2021 [84]; Martens et al., 2018 [85]
Ethical	Bhatti et al., 2021 [86]; Connally, 2021 [87]
Despotic	Khan et al., 2021 [88]; Wang et al., 2021 [89]
Toxic	Zaman et al., 2022 [90]
Autocratic	Zaman et al., 2021 [91]
Humble	Ali et al., 2020, 2021 [92–94]
Nontechnical	Kaminsky, 2012 [95]
Empowering	Capaldo et al., 2021 [37]; Nauman et al., 2022 [96]
Servant	Cleary-Hardy, 2021 [97]; Harwardt, 2020 [98]; Nauman et al., 2022 [4]
Supportive	Zaman et al., 2022 [99]
Knowledge-based	Latif et al., 2021 [84]; Mariam et al., 2022 [100]; Mubarak et al., 2021 [101]
Shared	Imam, 2021 [35]; Imam & Zaheer, 2021 [29]

As for shared leadership and its aforementioned counterparts or components, Table 1 shows that they have been investigated in only four papers, each focusing on specific project types. In [35], construction projects were examined, revealing that shared leadership plays a significant role in their success and meets individual psychological needs through member autonomy. Additionally, knowledge sharing moderates the relationship between shared leadership and autonomy. In [29], IT projects were considered, with knowledge sharing and cohesion as mediators and trust in the team as a moderator. The results show that shared leadership influences IT project success both directly, and through knowledge sharing and cohesion, with trust in the team interacting with both factors. Study [36] revealed that teamwork quality mediates the association between shared leadership and project success in (information system development projects). Furthermore, the results demonstrated that teamwork quality and project complexity moderate-mediate the relationship between shared leadership and project success. Capaldo et al. [37] investigated research projects and found that empowering leadership influences project success. Other literature items on leadership as a project success factor focus on other leadership styles.

This is an important research gap, as several studies have identified a positive relationship between shared leadership and team effectiveness and performance, although not explicitly within the project management context [38–44]. As mentioned in the Introduction, recent reports from practice clearly indicate that shared leadership may be significant for achieving project success, yet they do so without providing any scientific justification. For this reason, we hypothesised that *shared leadership is directly and positively related to project success* (H.1). Let us now proceed to describe our concretization of Box D (mediators).

### Potential mediators in the relationship between shared leadership and project success

The selection of team building, teamwork, justice, and individual engagement as mediators in our model is theoretically grounded in several well-established psychological frameworks. According to Social Exchange Theory [45], when leadership is distributed and perceived as fair and collaborative, team members are more likely to reciprocate through stronger relational ties, cooperation, and commitment—processes reflected in enhanced teamwork and perceptions of justice. Self-Determination Theory [46] posits that individuals are intrinsically motivated when their psychological needs for autonomy, competence, and relatedness are met. Shared leadership fosters autonomy and inclusion, leading to greater individual engagement and proactive involvement in team processes. Finally, Leader-Member Exchange Theory [47] emphasizes the importance of high-quality, trust-based exchanges between leaders and followers. In the context of shared leadership, these exchanges are not limited to hierarchical dyads but occur throughout the team, strengthening cohesion, mutual support, and role clarity—key outcomes of effective team building. Together, these mediators capture the interpersonal, motivational, and structural mechanisms through which shared leadership contributes to project success. Furthermore, based on conclusions drawn from the literature presented in Table 2, we believe that the selection of mediators is optimal for the current study.

**Table 2:** Potential mediators in the relationship between shared leadership and project success.

Category	Mediator	Definition	Hitherto results
Team-related characteristics	Team building	Facilitating group effectiveness, developing a sense of cohesion, and satisfying members’ needs [102].	Improves team outcomes, such as proactivity, creativity, satisfaction, effectiveness, and performance [43,102,103].
	Teamwork	Occurs when multiple interdependent individuals are effectively coordinated to achieve the desired goals [104]. Teamwork can be represented by team communication, collaboration, and cohesion [105,106].	Supports team performance [107,108], mediates the relationship between transformational leadership and project success [94,109,110].
Individual-related characteristics	Justice	Is understood as the employee’s perception of fairness in the workplace [111].	Has a positive impact on project governance [112], project success [113], project outcomes [114], citizenship behaviours of team members, and project performance [115].
	Individual engagement	Refers to an affective-motivational mindset of an individual, represented by vigour, dedication, and absorption [116].	Improves job and organisational performance [117,118], is a predictor of project success [4,67].

Table 2 can be concluded as follows:

i the set of mediators—team building, teamwork, justice, and individual engagement—has not yet been explored in the relationship between leadership and project success.
ii existing results referring to potential mediators show their positive mediational role in other aspects of effective and efficient project, organisation, or team management, suggesting they may also play a positive mediational role in the relationship between shared leadership and project success.

Team building and teamwork are considered team-related characteristics, while individual engagement and justice are viewed as individual-related characteristics, referring to the perception and behaviour of each project participant.

We thus hypothesised that *team building (H.2), teamwork (H.3), justice (H.4) and individual engagement (H.5) positively mediate the relationship between shared leadership and project success.*

### Conceptual model

**Fig 2:**
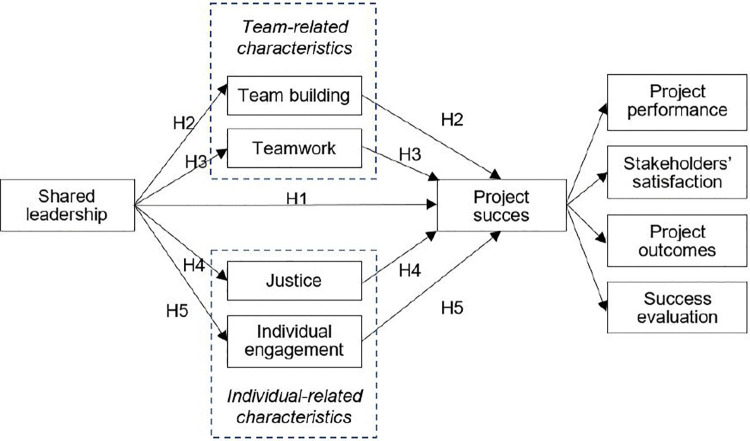
Conceptual model of the relationship between analysed variables shared leadership, team building, teamwork, justice, individual engagement, and project success.

The general model from Fig 1 has been concretised in the conceptual model in Fig 2.

Project success in Fig 2 will be measured:

- either by means of an aggregate of the four criteria categories (also called dimensions) on the left with equal weights, referred to as “aggregated project success,” or simply “project success” when there is no risk of ambiguity,
- or by one of the four criteria categories (dimensions) individually.

This mediation model was then tested and transformed using the data collected in a survey, which is described in the following section.

## Materials and methods

### Sample and data collection procedure

The study was conducted in Poland. The study recruitment period was from the 20th of January to the 3th of February 2022. Data suitable for analysis were obtained from 320 participants (158 women and 162 men), who were members in project teams. According to [48], our sample size was sufficient to ensure the external validity of the results and the generalizability of the conclusions. Participants ranged in age from 18 to 72 years (*M* = 40.5; *SD* = 12.4) and had been in active employment for an average of 16.7 years (*SD* = 11.6). Additional characteristics of the study participants, including the business sectors they represent, the type of projects they work on, the level of innovation and complexity of these projects, the type of project management they use, and their role in the projects, are included in Table 3.

**Table 3:** The sample characterictics.

		*n*	%
Participant’s role in the projects	Manager	73	22.8%
	Team member	241	75.3%
	Other	6	1.9%
Type of projects	Internal	156	48.8%
	External	115	35.9%
	Mixed	49	15.3%
Project innovativeness level	High or very high	103	32.2%
	Medium	174	54.4%
	Low or very low	43	13.4%
Project complexity level	High or very high	118	36.9%
	Medium	162	50.6%
	Low or very low	40	12.5%
Project management type	Traditional	222	69.4%
	Hybrid	68	21.3%
	Agile	25	7.8%
	Other	5	1.6%
Business sectors represented by the participants	Service and trade	86	26.9%
	Science	44	13.8%
	Industry	41	12.8%
	IT	39	12.2%
	Government administration	39	12.2%
	construction	38	11.9%
	other	34	10.6%

To avoid potential common method variance (CMV), we separated the measurement of the predictor and mediators by two weeks, collecting data at two different time points. This procedure of introducing a time interval between the measures allows us to assume that the participants’ ratings are relatively independent of each other [49]. Harman’s single-factor method was used to detect CMV. The results of the exploratory factor analysis showed only 35% of the variance for the single factor, which does not exceed the threshold value of 50% [50]. Thus, there was no substantial common method bias in this study.

Participation in the study was anonymous and voluntary. All participants provided their written consent to participate. Our study followed the ethical standards of the American Psychological Association and received approval from the Institutional Research Ethics Committee. The questionnaires were administered in Polish. Both the original Polish versions and the English translations (prepared for the purposes of this paper) were evaluated by five tenured team members proficient in both languages. The wording of some items in the Polish version (administered in the survey) was clarified to ensure maximum clarity, and the English translation was modified to convey the same message accurately.

### Measures

For each variable in Fig 2, we selected an appropriate scale. Additionally, for some variables, subscales were proposed to correspond to various dimensions of the concept represented by the given variable.

#### Project success.

Project success was measured using a 15-item questionnaire that included four subscales, corresponding to four project success dimensions, representing the four project criteria categories presented earlier. The items, identified based on the literature on project success, are listed in Table 4 and relate to four dimensions of project success: Project performance, (e.g., “the project was completed on time”), Stakeholders satisfaction, (e.g., “Clients/ end users were satisfied with project deliverables), Project outcomes, (e.g., “The deliverables of the project are used by its intended end users”), and Project evaluation, (e.g., “The project was successful (according to the respondent)”).

**Table 4:** Criteria of project success used in the survey.

Item no.	Project performance
1	The project was completed on time
2	The project was completed within to the budget
3	The project has achieved the desired level of quality
4	The project implemented the defined scope
5	Given the reason for which it was initiated, the project delivered the right solution
6	Project deliverables have been accepted by its end users in a short time
	**Stakeholders’ satisfaction**
1	Clients/ end users were satisfied with project deliverables
2	The project team members were satisfied with the process of project implementation
3	Project manager was satisfied with the process of project implementation
4	The key sponsors were satisfied with project deliverables
5	Project manager, and project team members were satisfied with project deliverables
	**Project outcomes**
1	The deliverables of the project are used by its intended end users.
2	The deliverables of the project are likely to be used over a longer period
3	It seems that as a result of the project, the organisation is better prepared for future operations
	**Success evaluation**
1	The project was successful (according to the respondent)

Each item was rated on a 5-point Likert-type scale from 1 (strongly disagree) to 5 (strongly agree). Internal reliability was satisfactory in the subscales (Cronbach’s α = .81 to .91), and in the total score (Cronbach’s α = .95).

#### Shared leadership.

Shared leadership was measured using the Shared Leadership Questionnaire [33,34], which consists of 26 items outlined in six subscales: transformational (e.g., “My team members showed enthusiasm for my efforts”), transactional (e.g., “My team members gave me positive feedback when I performed well”), participative (e.g., “My team members and I worked together to decide what my performance goals should be”), individual-related empowering (e.g., “My team members encouraged me to learn new things”), team-related empowering (e.g., “My team members encouraged me to work together with other individuals who are part of the team”), and aversive leadership, which was reversely coded (e.g., “My team members could be quite intimidating”). All items are listed in S1 and S2 Files. A 5-point Likert scale was used to rate each item from 1 (strongly disagree) to 5 (strongly agree). Cronbach’s alpha for the subscales ranged from .73 to .82, and the total score for shared leadership was .91.

#### Team building.

Team building was assessed using a 17-item questionnaire developed by [51]. It consists of four subscales related to broad areas of team-building processes: goal setting (e.g., “Setting project goals on a participatory basis by the team”), interpersonal relations (e.g., “Conducting training programs on communication skills for the project team”), role clarification (e.g., “Clarifying role expectations for each team member”), and problem-solving (e.g., “Ensuring the participation of the project team(s) in designing action plans to solve task-related problems of the project”). All items are listed in S1 and S2 Files. Each item was scored on a five-point Likert scale from 1 (never) to 5 (always). Cronbach’s α values for the subscales ranged from .78 to .89, and the total score for team building was .95.

#### Teamwork.

We measured teamwork based on the conceptualization by Barrick et al. [52], using 25 items and three subscales: team communication (e.g., “There was frequent communication within the team”), team cohesion (e.g., “It was important to the members of our team to be part of this project”), and team collaboration (e.g., “We achieved project goals collectively”). All items are listed in S1 and S2 Files. Participants rated the items on a five-point scale, from 1 (strongly disagree) to 5 (strongly agree). The Cronbach’s α values for the subscales ranged from .81 to .93.

#### Justice.

Justice was assessed using the four-item justice subscale of the Copenhagen Psychosocial Questionnaire [53]. Each item was rated on a five-point scale, from 1 (to a very small extent) to 5 (to a very large extent). All items are listed in S1 and S2 Files. An example item is “Team members were recognized when they performed well.” Cronbach’s α was .82.

#### Individual engagement.

Individual engagement was measured using a six-item scale representing physical, cognitive, and emotional aspects [54]. All items are listed in S1 and S2 Files. An example item from this scale is “I really ‘throw’ myself into my work.” Items were rated on a five-point scale, from 1 (to a very small extent) to 5 (to a very large extent). The Cronbach’s α for this scale was .87.

### Data analysis

Correlation and mediation analyses using structural equation modelling (SEM) were performed [55]. Following Kline’s [56] recommendations, SEM was utilized to identify, evaluate, and revise the measurement and structural model. The measurement model had a very good fit to the data: the root mean square error of approximation (RMSEA) and the standardized root mean square residual (SRMR) were below .05, and the values of the adjusted goodness of fit index (AGFI), comparative fit index (CFI), and Tucker-Lewis fit index (TLI) were above .90. To determine the significance of the indirect effect coefficient, the 10,000 samples bootstrapping procedure was used. All continuous variables were standardized to z scores before analysis. The analyses were carried out using IBM SPSS 25.0, and the SEM models were analysed using AMOS 25.0 software.

## Results

### Preliminary analysis

Means, standard deviations, and correlations between the analysed variables are in Table 5.

**Table 5:** Means, standard deviations, and correlations between analysed variables.

	*M*	*SD*	1	2	3	4	5	6	7	8	9	10	11	12	13	14	15	16	17	18	19
*Shared leadership*	*–*	*–*																			
1 Transformational	19.86	3.90	–																		
2 Transactional	14.02	2.77	.52^**^	–																	
3 Participative	14.32	3.09	.48^**^	.67^**^	–																
4 Empowerment (individual)	13.89	3.14	.55^**^	.66^**^	.62^**^	–															
5 Empowerment (team)	15.13	2.74	.49^**^	.64^**^	.54^**^	.55^**^	–														
6 Aversive	8.85	3.26	.20^**^	−.11^*^	−.10	.04	−.19^**^	–													
*Project success*																					
7 Project performance	24.48	3.86	.22^**^	.42^**^	.36^**^	.27^**^	.40^**^	−.29^**^	–												
8 Stakeholders’ satisfaction	20.53	3.29	.23^**^	.44^**^	.35^**^	.33^**^	.43^**^	−.27^**^	.84^**^	–											
9 Project outcomes	12.24	2.09	.22^**^	.37^**^	.28^**^	.25^**^	.31^**^	−.24^**^	.75^**^	.73^**^	–										
10 Success evaluation	4.21	.82	.18^**^	.36^**^	.31^**^	.24^**^	.30^**^	−.25^**^	.74^**^	.77^**^	.69^**^	–									
*Teamwork*																					
11 Team communication	39.03	5.93	.43^**^	.66^**^	.52^**^	.48^**^	.63^**^	−.31^**^	.54^**^	.57^**^	.45^**^	.50^**^	–								
12 Team cohesion	38.95	6.67	.43^**^	.72^**^	.54^**^	.51^**^	.56^**^	−.28^**^	.53^**^	.55^**^	.46^**^	.51^**^	.88^**^	–							
13 Team collaboration	19.31	3.29	.38^**^	.65^**^	.53^**^	.45^**^	.54^**^	−.27^**^	.53^**^	.54^**^	.43^**^	.46^**^	.85^**^	.85^**^	–						
*Team-building*																					
14 Goal setting	15.77	2.98	.36^**^	.57^**^	.43^**^	.39^**^	.50^**^	−.26^**^	.55^**^	.55^**^	.46^**^	.48^**^	.74^**^	.73^**^	.71^**^	–					
15 Interpersonal relations	14.37	3.27	.40^**^	.54^**^	.41^**^	.41^**^	.42^**^	−.05	.41^**^	.39^**^	.32^**^	.35^**^	.60^**^	.63^**^	.57^**^	.68^**^	–				
16 Role clarification	16.04	2.93	.28^**^	.48^**^	.35^**^	.32^**^	.49^**^	−.25^**^	.54^**^	.54^**^	.47^**^	.50^**^	.72^**^	.72^**^	.66^**^	.84^**^	.63^**^	–			
17 Problem solving	19.07	3.81	.38^**^	.58^**^	.45^**^	.45^**^	.50^**^	−.19^**^	.48^**^	.52^**^	.47^**^	.47^**^	.74^**^	.74^**^	.69^**^	.84^**^	.73^**^	.77^**^	–		
*Individual engagement*																					
18 Individual engagement	23.62	4.05	.34^**^	.46^**^	.37^**^	.41^**^	.48^**^	−.18^**^	.47^**^	.51^**^	.46^**^	.48^**^	.61^**^	.62^**^	.59^**^	.52^**^	.42^**^	.51^**^	.54^**^	–	
*Justice*																					
19 Justice	15.76	2.88	.31^**^	.58^**^	.43^**^	.42^**^	.47^**^	−.28^**^	.51^**^	.52^**^	.42^**^	.44^**^	.72^**^	.75^**^	.70^**^	.80^**^	.68^**^	.80^**^	.77^**^	.49^**^	–

Note: * *p* *<* .05*,* ^**^ *p* < .01.

Except for the correlations between aversive leadership and participative leadership, individual empowerment, and interpersonal relations, the remaining correlations between the studied variables were significant and ranged from −.11 to .88. All dimensions of shared leadership were significantly and moderately correlated with all dimensions of project success, as well as with aggregated project success (with the four dimensions weighted equally). Moderate to strong significant correlations were also observed between shared leadership dimensions and teamwork dimensions, team-building dimensions, individual engagement, and justice. Aversive leadership was significantly negatively correlated with all variables except for empowerment (team) and individual leadership, and the interpersonal relations dimension of team building. Individual dimensions of project success were significantly and moderately associated with the dimensions of teamwork, team building, individual engagement, and justice.

### Shared leadership as factor of aggregated project success

#### Results for the conceptual model.

First, we tested the direct relationship between shared leadership and project success with the project success criteria dimensions aggregated with equal weights (i.e., aggregated project success, or simply project success), in order to verify hypothesis H1. The total effect of shared leadership on project success was statistically significant, *b =* .69, *se* = .07, β = .53, *p* < .001, and explained 29% of the variance. The SEM model which tested the relationship between two latent variables, shared leadership and project success, was well fitted to the data, χ² = 55.17, df = 29, χ²/df = 2.01, *p* = .001, RMSEA = .056, GFI = .97, AGFI = .94, CFI = .99, TLI = .98, SRMR = .055. This means that shared leadership was directly related to project success, measured by an aggregation, with equal weights, of success dimensions referring to project performance, stakeholders’ satisfaction, project outcomes, and subjective success evaluation. This supports H1 for the project success definition selected here.

Next, we examined the indirect associations of shared leadership with overall project success, mediated in parallel by teamwork, team building, justice, and project engagement (Fig 2). However, after including the mediators, the effect of shared leadership on project success became statistically nonsignificant (b = −.04, boot se = .32, β = −.02, 95% CI [−.64, .65]) and explained 46% of the variance. The overall model resulted in unsatisfactory fit indices [57], χ² = 705.76, df = 145, χ²/df = 4.87, p < .001, RMSEA = .110, GFI = .77, AGFI = .70, CFI = .90, TLI = .88, SRMR = .069.

#### Building the final model.

**Fig 3:**
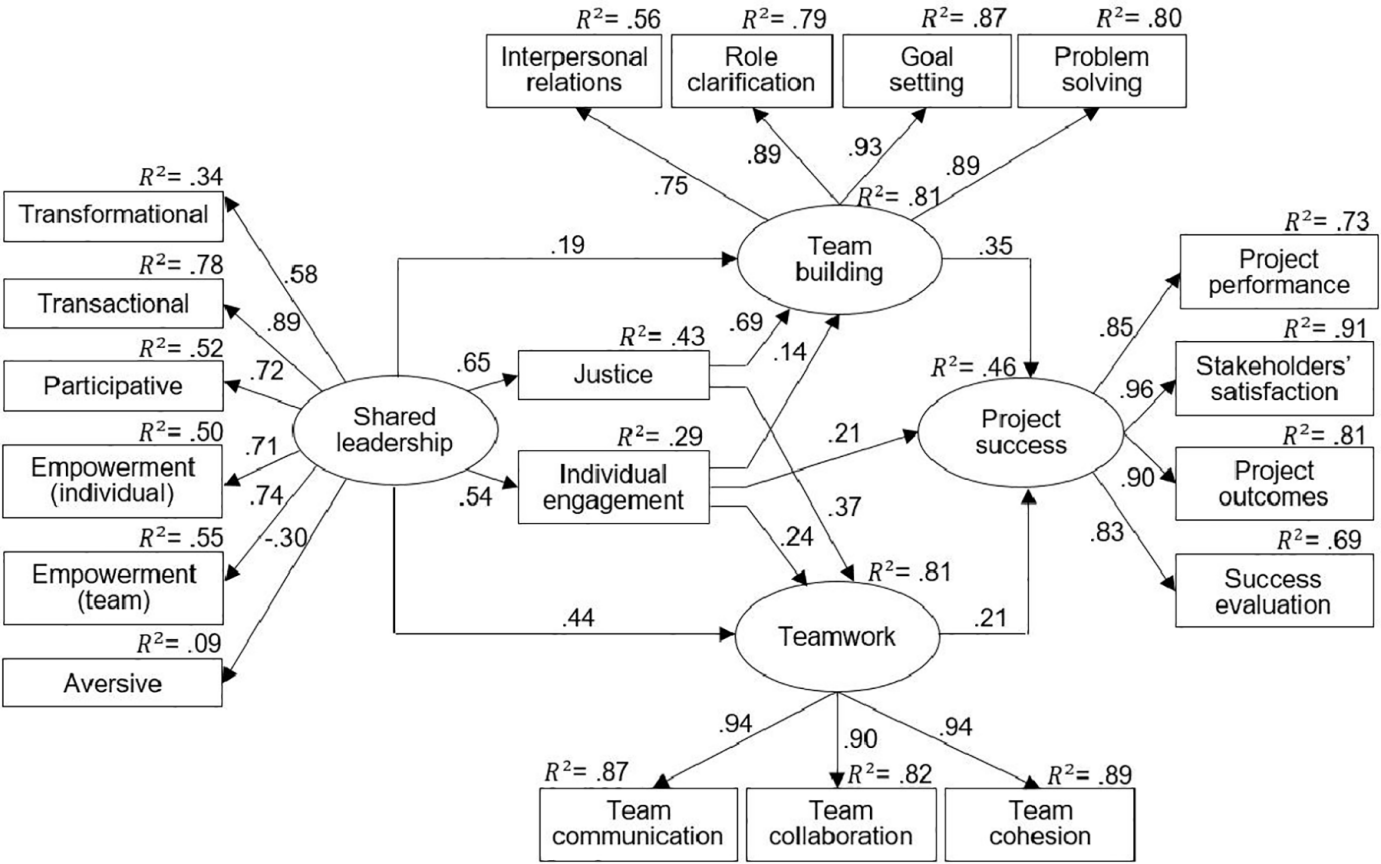
Final model (FM0) of the relationship between shared leadership, individual engagement, team building, teamwork, and project success. *Note.* Regression coefficient β for all paths are statistically significant.

The conceptual model from Fig 2 was then stepwise modified by excluding insignificant paths related to parallel mediation, including new paths representing serial mediations, and adding covariances between measurement errors. This process resulted in the model shown in Fig 3, from now on referred to as Final Model 0 (FM0), which was well-fitted to the data. All fit indices exceeded the recommended cut-off criteria [57], χ² = 225.04, df = 126, χ²/df = 1.79, *p* < .001, RMSEA = .051, GFI = .93, AGFI = .90, CFI = .98, TLI = .97, SRMR = .041. All paths turned out to be statistically significant (see Fig 3).

#### Results for the final model.

Model FM0 revealed statistically significant mediation effects. There were three significant simple indirect effects, representing the parallel mediational role of individual engagement, team building, and teamwork in the relationship between shared leadership and project success. In addition, four serial mediation effects between shared leadership and project success were statistically significant: through individual engagement and team building, through individual engagement and teamwork, through justice and team building, and through justice and teamwork. This is presented in the first column of Table 5. If the confidence interval (CI) does not contain zero, the indirect effect is statistically significant. Altogether, shared leadership, controlling for individual-related (justice, individual engagement) and team-related mediators (team building, teamwork), explained 46% of the variance in project success.

### Shared leadership as factor of project success defined as individual success dimensions

**Fig 4:**
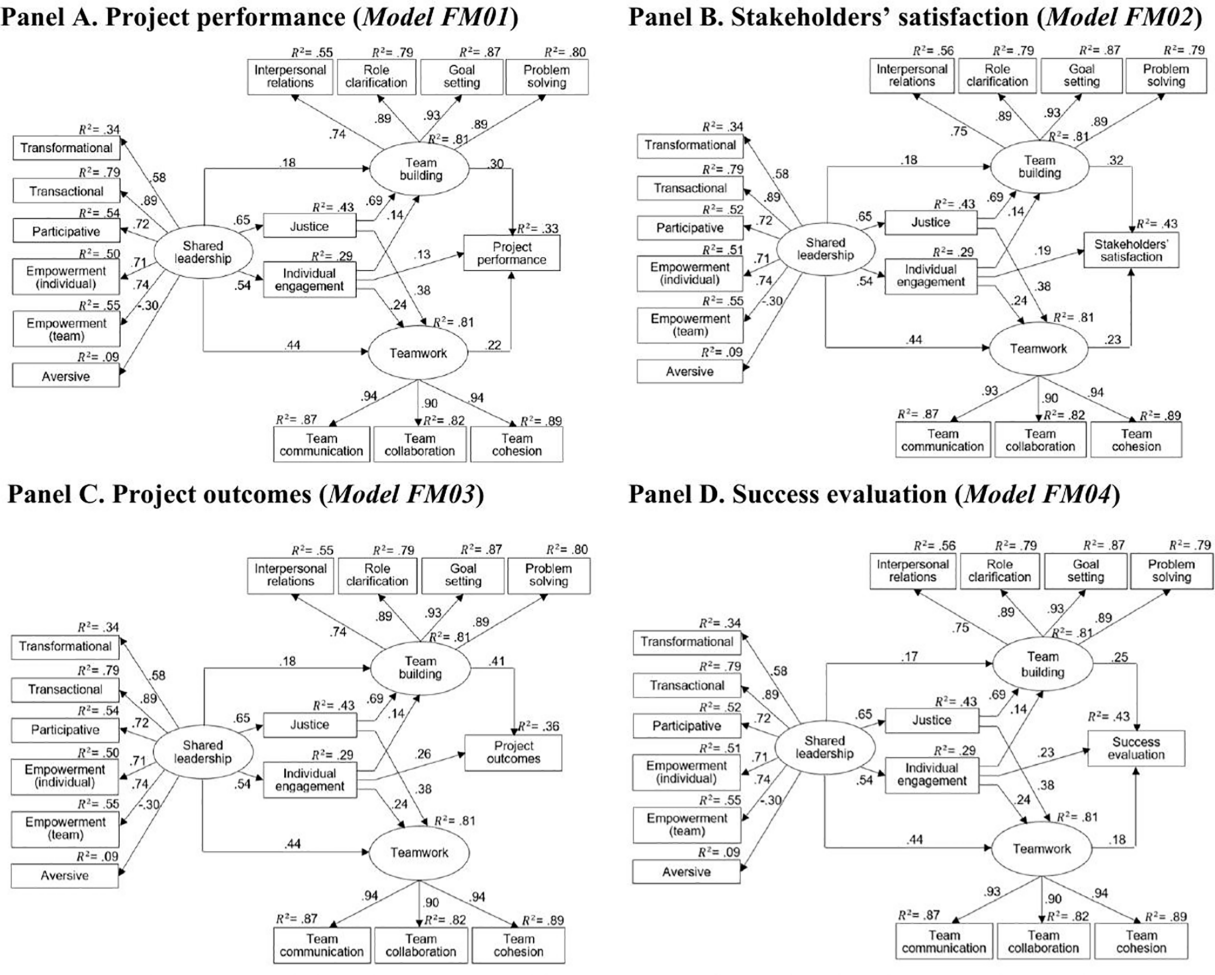
Mediation model of the relationships between shared leadership, justice, individual engagement, team building, teamwork, and each of project success criteria. *Note.* Regression coefficient β for all paths are statistically significant*. Model 6:* χ^2^ = 180.41, df = 82, χ^2^/df = 2.20, RMSEA = 0.061, AGFI = 0.89, CFI = 0.98, TLI = 0.97, SRMR = 0.069. *Model 7:* χ^2^ = 159.99, df = 82, χ^2^/df = 1.95, RMSEA = 0.055, AGFI = 0.90, CFI = 0.98, TLI = 0.97, SRMR = 0.039. *Model 8:* χ^2^ = 168.06, df = 83, χ^2^/df = 2.02, RMSEA = 0.057, AGFI = 0.90, CFI = 0.98, TLI = 0.97, SRMR = 0.040. *Model 9:* χ^2^ = 171.78, df = 83, χ^2^/df = 2.07, RMSEA = 0.058, AGFI = 0.90, CFI = 0.98, TLI = 0.97, SRMR = 0.041.

To expand the knowledge on the mediating role of individual- and team-related factors in the relationship between shared leadership and project success, we also tested models of the relationships between shared leadership and four project success dimensions individually: project performance (Model FM1), stakeholders’ satisfaction (Model FM2), project outcomes (Model FM3), and subjective project success evaluation (Model FM4) (see Fig 4). The models were constructed by removing three project success dimensions each time.

The indirect effects in Models FM1-FM4 are presented in Table 6.

**Table 6:** Indirect effects in the tested SEM models for (aggregated) project success, and for individual dimensions of project success.

Indirect effects	Aggregated project success (FM0)		Project performance (FM1)		Stakeholders’ satisfaction (FM2)		Project outcomes (FM3)		Success evaluation (FM4)	
	Effect(*se*)	95% CI	Effect(*se*)	95% CI	Effect(*se*)	95% CI	Effect(*se*)	95% CI	Effect(*se*)	95% CI
Shared leadership → team building →	.09(.04)^**^	[.03,.20]	.09(.05)^**^	[.02,.22]	.08(.04)^**^	[.03,.19]	.13(.05)^**^	[.04,.24]	.07(.01)^**^	[.01,.04]
Shared leadership → teamwork→→	.13(.07)^*^	[.01,.27]	.16(.10)^†^	[-.01,.37]	.15(.07)^*^	[.01,.29]			.13(.01)	[-.03,.32]
Shared leadership → individual engagement →	.16(.06)^**^	[.05,.29]	.12(.07)^†^	[-.01,.27]	.15(.04)^*^	[.04,.28]	.24(.07)^***^	[.10,.40]	.21(.01)^**^	[.07,.38]
Shared leadership → justice → team building →	.22(.07)^**^	[.10,.39]	.23(.09)^**^	[.07,.43]	.21(.07)^**^	[.08,.38]	.32(.07)^***^	[.17,.48]	.19(.02)^**^	[.01,.38]
Shared leadership → justice → teamwork →	.07(.04)^*^	[.01,.17]	.09(.05)^†^	[-.01,.22]	.08(.06)^*^	[.01,.18]			.07(.04)^†^	[-.02,.20]
Shared leadership → individual engagement → team building →	.04(.02)^**^	[.01,.08]	.04(.02)^**^	[.01,.09]	.04(.02)^**^	[.01,.09]	.05(.02)^***^	[.02,.10]	.03(.01)^**^	[.01,.08]
Shared leadership → individual engagement → teamwork →	.04(.02)^*^	[.01,.09]	.05(.03)^*^	[.01,.13]	.04(.02)^*^	[.01,.10]			.04(.01)^†^	[-.01,.11]

Note: † *p <* .1, ^*^ *p <* .05, ^**^ *p <* .01, ^***^ *p <* .001.

The first column of Table 6 (Model FM0) was discussed in the previous subsection. Model FM1 explained 33% of the variance, Model FM2 explained 43%, Model FM3 explained 36%, and Model FM4 explained 33%. In terms of mediating effects, team building showed a significant simple mediation effect for every dimension of project success: project performance, stakeholders’ satisfaction, project outcomes, and subjective success evaluation. The simple mediating effect of individual engagement was significant for all dimensions, although marginally for project performance. This effect was stronger for project outcomes and subjective success evaluation than for aggregated project success. Teamwork showed a significant mediation effect only for stakeholders’ satisfaction, a marginally significant effect for project performance, and a non-significant effect for project outcomes and subjective success evaluation. There were no significant mediation effects of justice for any of the project success dimensions.

## Discussion

Our study aimed to explore the mechanism underlying the relationship between shared leadership and project success. Therefore, we examined the mediating role of team-related characteristics (i.e., team building and teamwork) and individual-related characteristics (i.e., individual engagement and justice, understood as fairness perception) in this relationship. In our study, project success was considered a multidimensional construct, based on project performance, stakeholders’ satisfaction, project outcomes, and subjective evaluation of project success [4,12,51].

### Theoretical implications

The findings of the paper address the following identified research gaps:

- The relationship between shared leadership and project success had been investigated in only four studies [29,35–37], all focusing on highly specific projects, where it was found to be significant. Beyond this, no further insights into the relationship had been established.
- The mediating roles of team building, teamwork, justice, and individual engagement in the relationship between leadership and project success remained unexplored, despite these factors having demonstrated significance in other areas of management.
- The project success criteria employed in our study had not previously been examined in relation to project success.

This paper contributes to all three of these aspects. These gaps correspond with ongoing calls for more refined models in project leadership research. Turner and Müller [8] highlighted that project success research had largely overlooked the role of leadership style and called for integrating leadership theory into this domain. More recent studies have advanced this by showing that the leadership–success link is not straightforward but mediated by processes such as team-building and collaboration [58,59].

To start with, we positively verified hypothesis H1, which posited a direct relationship between shared leadership and aggregated project success, composed of the four success criteria listed above, equally weighted. This finding (that shared leadership is directly related to project success) is consistent with previous studies [29,35]. All project team members should recognize that participation in activities that improve joint decision-making and sharing responsibility affects other team members, boosting team effectiveness and ultimately supporting joint success, as well as improving cooperation in decision-making and responsibility sharing [34,60].

The effect of shared leadership on aggregated project success was mediated in parallel by individual engagement, team building, and teamwork, thereby supporting H2, H3, and H5. However, justice did not mediate this relationship, and thus H4 was not verified. The strongest simple mediating effect in the relationship between shared leadership and aggregated project success was observed for individual engagement, which is an important indication for project managers. It is possible that sharing the responsibility of motivating, inspiring, or coaching one another increases the willingness of individual project team members to engage in behaviours that enhance the chances of achieving project success. Teamwork had the second strongest mediating effect in this relationship. Presumably, components of shared leadership, most likely team empowerment, improve the quality of collaborative work within the project team, which in turn increases the chances of project success. The mediating effect of team building was lower than that of teamwork.

We also found serial mediating effects of shared leadership on aggregated project success through chains of mediators. First, shared leadership positively affected individual engagement, which, in turn, increased teamwork and subsequently increased the chances of project success. Similarly, shared leadership increased individual engagement, which positively impacted team building, thereby improving the project’s chances for success. We can thus conclude that shared leadership, through its positive effect on individual engagement, enables team-building efforts and promotes project success.

Other serial mediating effects shed light on the role of justice in the tested relationships. Although justice did not have a strong direct impact on project success (H4 was not verified), it played an important indirect role. Shared leadership helps build a sense of justice in the team – mainly through participative decision-making and empowerment. Justice, in turn, improves teamwork and team building, which then boosts project success. The strongest effect was seen when justice led to better team building.

In summary, the simple mediation effects identified imply that project managers, while striving to achieve success in projects, should focus on team members, strengthening their individual engagement while simultaneously considering the proper formation of the team and the rules governing teamwork. All the serial mediation relationships between shared leadership and aggregated project success revealed the following mechanism: shared leadership first affected the individual motivation of project members (engagement or justice), which enhanced team processes (team building or teamwork), and consequently increased the chances of project success. According to the model of collective effort by [61], project members are motivated to work harder when they believe their efforts contribute to collective productivity and overall project performance.

We tested the respective simple and serial mediating effects not only for aggregated project success but also for each project success dimension (criteria category) individually. The results showed that the mechanism explaining the effect of shared leadership on project success may depend on the approach taken by the decision-maker in understanding project success. The main differences are summarized in Table 7, as implied by the results presented in Table 6.

**Table 7:** Effect of Shared Leadership on Project Success for its various definitions.

Effect	PS	PP	SS	PO	SE
Shared leadership → team building →	3	3	3	3	3
Shared leadership → teamwork →	2	1	2	0	0
Shared leadership → individual engagement →	3	1	2	4	3
Shared leadership → justice → team building →	3	3	3	4	3
Shared leadership → justice → teamwork →	2	1	2	0	1
Shared leadership → individual engagement → team building →	3	3	3	4	3
Shared leadership → individual engagement → teamwork →	2	2	2	0	1

Note: PS – Aggregated project success; PP – Project performance; SS – Stakeholders’ satisfaction; PO – Project outcomes; SE – Success evaluation; (0 – insignificant effect, 4 – very significant effect).

Table 6 may be a source of valuable information on which strategy to choose when using shared leadership in project management. For example, if project performance (PP: time, cost, quality, scope) had the highest weight in the evaluation of project success (such as in government administration units [62], using shared leadership to increase teamwork or individual engagement would not be the most efficient strategies. Instead, increasing team building directly, or through justice or individual engagement, are the recommended strategies for this understanding of project success. If the most important project success dimension were the project outcomes (PO: in the sense of the project’s effect over a longer period), influencing teamwork through shared leadership, either directly or indirectly, would not be the appropriate strategy; team building and individual engagement would be the most significant mediators, and the selected strategy should be based on them. Analogous conclusions and thus practical implications for project management can be drawn for each of the five project success definitions (the aggregated project success PS, and those based on each of the four dimensions individually).

The most problematic success dimension is the subjective evaluation of project success (SE). This dimension strongly depends on the respondent or decision maker. However, in view of the difference between column SE and the other columns in Table 6, our results indicate an important issue: the approach taken in project management to satisfy more objective project success criteria may not coincide with the approach needed to ensure that individual project stakeholders perceive the project as a success.

As we mentioned in the Introduction, key project stakeholders may not be unanimous on the definition of project success. For example, the project manager might prioritize project performance (PP), while higher management of the organisation might consider stakeholders’ satisfaction (SS) more important. In such a case, we can analyse Table 7 horizontally, taking into account only the columns representing the project success definitions selected by our stakeholders, and choose a strategy for achieving project success that constitutes a compromise among the selected definitions. In the aforementioned example, columns PP and SS would be analysed horizontally. We would find that using shared leadership to increase team building, whether directly or through justice or individual engagement, would be the most efficient strategies. If the project success definitions chosen by stakeholders were, for example, project performance (PP) and project outcomes (PO), the best approach would be to use shared leadership to increase team building directly or through individual engagement.

As with any multicriteria decision, the choice of strategy on how to use shared leadership to increase the chances of project success, when it is understood in various ways, may not be unambiguous. In the case of the project success definitions considered in this paper, the strategy of using shared leadership to increase team building through individual engagement seems to be the optimal one, especially if there is indecisiveness among stakeholders or a lack of clear information from them.

### Managerial implications

In view of the importance of shared leadership, understood as the “active and intentional shifting of the role of leader to the group, as necessitated by the environment or circumstances in which the group operates,” companies should consider changes in their recruitment, training, and motivation policies to build cultures that support the efficient implementation of shared leadership and its components or counterparts, such as transactional, transformational, or empowering leadership.

Another important implication for managers is to avoid introducing a static approach to project management in their organisations. The aforementioned definition of shared leadership highlights the importance of reacting to the current environment or circumstances. Project team members and leaders should be aware of this issue, given the dynamic nature of projects and their environments today. This involves adapting the concrete implementation of shared leadership to the current project success definition, or definitions, specific to the project or project stakeholders.

Recruitment, training, and motivation policies should not only include shared leadership and its individual dimensions but also the mediators (and their dimensions) in the relationship between shared leadership and project success, relevant to the project success definitions likely to be adopted in the organisation’s future projects. The measures for the mediators, listed in S1 and S2 Files, provide a ready-made curriculum for training sessions. In these training sessions, competencies in applying shared leadership to support the selected mediators should be imparted. This constitutes a challenge for all organisations, including those providing training to project managers and team members. Concrete examples of steps to be taken in order to implement shared leadership might include:

- rotating roles (instead of a single leader making all decisions, assigning different team members to lead various aspects of the project);
- delegated decision-making (empowering individuals by delegating decisions to the most knowledgeable person in the respective area);
- peer-to-peer mentoring (establishing a system where team members mentor each other on different aspects of the project);
- collaborative tools (utilizing project management and collaboration tools, like Trello, Asana, or Miro) where all team members can contribute ideas, track progress, and comment on decisions.

Similar examples can be proposed for the implementation or enhancement of the mediators. For instance, team building would be encouraged among others by:

- periodic team-building activities that go beyond project tasks;
- workshops where team members explain their roles and challenges to one another;
- implementing systems for peer recognition, so that team members can highlight each other’s contributions.

### Limitations and future research directions

As indicated by the literature, mediation models have some limitations and should be interpreted with caution [63]. Most notably, the cross-sectional design of this study precludes any conclusions about causality. While we tested theoretically informed mediation patterns, we were not able to determine which variable came first, so no directional or causal conclusions can be drawn. The associations identified may reflect correlation rather than influence, and alternative explanations are plausible. Definitive claims about mediation or causal mechanisms would require longitudinal or experimental research designs capable of capturing changes over time. Additionally, reliance on self-reported data introduces potential biases, such as social desirability effects and common method variance. To improve the validity and generalizability of future studies, the use of complementary data sources—such as peer or supervisor ratings, behavioural observations, or network-based assessments—should be considered. Moreover, the used measure of project success require further testing of reliability and validity.

Secondly, different project success factors contribute to project success at different stages of the project. Additionally, conclusions might vary for different stages of project preparation, corresponding to various starting points in Fig 2. The advised strategy might differ if no preparation steps for the project have been undertaken so far (the lower left corner of the polygon) compared to when the current (yet unfinished) project plan places the project, for example, close to point A, but project success criterion S_2_ has turned out to be most significant. Due to our cross-sectional study design, we were unable to explore these dynamics. Therefore, future research should address these aspects.

Another element to which mediation models are highly sensitive is the measurement method—in our case, the questionnaires. For example, shared leadership can be measured differently, for example, by directly asking about the membership roles assumed by project team members. This approach would be in line with the definition of shared leadership. As presented earlier, the measurement of project success can also be implemented in various ways. Therefore, other models should be explored in future research.

The need for other models also arises from the problem of weights, mentioned earlier in the context of project success. This issue, however, also pertains to the dimensions of shared leadership and the mediators: it is possible to consider and implement all these notions with shifting emphasis on individual dimensions, thus with different weights. Therefore, to confirm our findings, future research should explore other models where dimension weights for all the variables involved can be selected by the decision maker.

The need for exploring additional models also arises from the fact that our research did not consider any moderators, which are defined as variables that affect the direction and/or strength of the analysed relationships. The importance of these variables in the context considered here is clearly demonstrated in [64] and [65] where the moderating role of several project-level, producer-level, firm-level, and country-level variables is examined in a similar context. In [29] trust is considered as a moderator of the relationship between shared leadership and project success. These research directions should be further explored for shared leadership and its role as a project success factor. Furthermore, future studies could test reversed mediation, for example by asking whether project success fosters shared leadership, or they could use social network analysis to study leadership distribution.

Another issue is the problem of shared leadership implementation. Simply deciding to apply shared leadership is not enough to make it effective, as indicated in [66]. Without successful shared leadership application, all the conclusions drawn in this and similar papers would be worthless. In this context, applying a nuanced weighting of various shared leadership dimensions in our and similar models might be useful, with the weights depending on the actual state of shared leadership implementation.

The last issue is that we collected data from project members in Poland, so the generalizability beyond this cultural context is limited. For example, organisational justice is interpreted and perceived differently in different cultures. This highlights the need for research into the impact of cultural differences on the relationship between shared leadership perceptions, justice and project success [67], as well as other aspects discussed in this paper. Cross-cultural investigations could reveal how cultural norms surrounding hierarchy, power distance, and collectivism shape leaders’ behaviors and employees’ responses to shared leadership and perceived fairness. Such insights would not only enrich leadership theory but also inform culturally responsive practices in international project management and global organizations. Future research should also extend this inquiry to other dimensions discussed in this paper and beyond, including risk-taking, adaptability, and psychological safety, which may similarly vary across cultural settings and impact organizational outcomes in nuanced ways. To enhance the generalizability of our findings, future studies should examine whether the observed relationships vary across different contextual factors, such as project complexity, duration, industry sector, and management methodology (e.g., traditional vs. agile). Such investigations would provide deeper insight into how shared leadership dynamics and their mediators function under varying organizational and project-specific conditions.

In our opinion, the most important future research directions should focus on yet unexplored leadership styles, mediators, and moderators that might influence the chances of project success, as well as on broader project contexts, such as different types of organisations, cultures, and societies. The impact of shared leadership may be influenced by moderating factors such as project complexity, team size, or organizational culture—variables not explored in this study, but which offer promising directions for future research. In addition, future research should employ specific project success criteria, considering various profiles of decision makers and project types, and include sensitivity analyses. Such research would help find a compromise among stakeholders on the definition of project success and determine whether a chosen strategy for project leadership is appropriate in a given context.

## Conclusions

Improving knowledge about the aspects that influence project success is of great importance for project-based organisations. This study makes a significant contribution to both project management theory and professional practice. From a theoretical perspective, our research shows that shared leadership has a positive and significant impact on project success. This is very good news for project leaders. As pointed out by [60], with the complexity and ambiguity linked to projects nowadays, a single leader is unlikely to possess all the skills and attributes required to lead projects effectively on their own. Our findings show that they can share the burden of project management with project team members. We have demonstrated that shared leadership has both direct and indirect effects on project success when it is defined as a construct of four dimensions (project performance, stakeholders’ satisfaction, project outcomes, and subjective evaluation of project success) or in terms of each dimension separately.

We confirmed a parallel simple mediation role of team-related factors (i.e., team building and teamwork) and individual engagement. Furthermore, we revealed serial mediating effects of shared leadership on project success through chains of individual- and team-related variables: individual engagement and team building, justice (fairness perception) and team building, individual engagement and teamwork, and justice and teamwork. We also proposed several strategies to be used by project managers, depending on their approach to project success. Our findings may represent complex and important mechanisms that explain the relationship between shared leadership and project success. Therefore, project-oriented organisations should promote a shared leadership style among project managers and teams to benefit from our findings and the developed management strategies.

Additionally, we identified innovative research possibilities that would assist researchers and practitioners in better understanding the factors influencing project success, which is universally desired but often interpreted differently by individuals. From a practical standpoint, the study offers valuable insights for project leaders and HR managers. It demonstrates that effective implementation of shared leadership requires the intentional development of team members’ engagement and the cultivation of high-quality communication, collaboration, and team-building processes. Moreover, the article provides practitioners with analytical tools and strategic recommendations for selecting appropriate leadership approaches based on how project success is defined by key stakeholders. The insights presented can serve as a foundation for training programs for project managers and inspire the development of organizational cultures that support shared leadership.

## Supporting information

S1 File Measurement instruments used in the study. (DOCX)

S2 File Measurement instruments used in the study (in Polish). (DOCX)

S3 File Database. (XLS)
