# Supplementary material for: Shared leadership and project success: The mediational role of individual- and team-related factors
Source: PLoS One. 2026 Feb 13;21(2):e0342268. doi: 10.1371/journal.pone.0342268 (PMC12904471; doi:10.1371/journal.pone.0342268)
Supplement: S1 File — (DOCX) [file pone.0342268.s001.docx]

**S1 File. Measurement instruments used in the study**

## Measure items for Shared Leadership

All the questions were clearly related to the respondent's last project, starting with the words: “In my last project…”

#### Transformational Leadership

1. My team members provided a clear vision of who and what our team was.
2. My team members were driven by higher purposes or ideas.
3. My team members showed enthusiasm for my efforts.
4. My team members encouraged me to rethink ideas that had never been questioned before.
5. My team members sought a broad range of perspectives when solving problems.
6. My team members encouraged me to go above and beyond what was normally expected (e.g., extra effort).

#### Transactional Leadership

1. My team members and I had clear agreements and stuck to them when we worked together.
2. When I performed well, my team members recommended more compensation.
3. My team members gave me positive feedback when I performed well.
4. My team members gave me special recognition when my work performance was especially good.

#### Participative Leadership

1. My team members decided my performance goals with me.
2. My team members and I worked together to decide what my performance goals should be.
3. My team members and I sat down together and reached an agreement on my performance goals.
4. My team members worked with me to develop my performance goals.

#### Empowering Leadership (Individual-Related)

1. My team members encouraged me to search for solutions to my problems without supervision.
2. My team members urged me to assume responsibilities on my own.
3. My team members encouraged me to learn new things.
4. My team members encouraged me to give myself a pat on the back when I met a new challenge.

#### Empowering Leadership (Team-Related)

1. My team members encouraged me to work together with other individuals who are part of the team.
2. My team members advised me to coordinate my efforts with other individuals who are part of the team.
3. My team members urged me to work as a team with other individuals who were part of the team.
4. My team members expected that the collaboration with other members of the team would work well.

#### Aversive Leadership

1. My team members tried to influence me through threats and intimidation.
2. I felt intimidated by my team members’ behavior.
3. My team members could be quite intimidating.
4. When my work was not up to par, my team members pointed it out to me.

## Measure items for Team Building

All the questions were clearly related to the respondent's last project, starting with the words: “In my last project the following phenomena actually occurred:”

#### Goal Setting

1. Setting project goals on a participatory basis by the team.
2. Involving project team members in action planning to identify ways to achieve project goals.
3. Making the basic goals of the project clear to the project team.
4. Letting the project team receive timely feedback on performance in relation to the goals of the project.

#### Interpersonal Relations

1. Encouraging team members to meet with each other during the project.
2. Discussing conflicts among project team members frankly.
3. Discussing relationships among project members frankly.
4. Conducting training programs on communication skills for the project team.
5. Creating opportunities for sharing feelings among the project team.

#### Role Clarification

1. Clarifying role expectations for each team member.
2. Giving information about the shared responsibilities of team members.
3. Making project norms familiar to each team member.

#### Problem Solving

1. Involving the project team(s) in identifying task-related problems.
2. Involving the project team(s) in generating ideas concerning the causes of task-related problems.
3. Ensuring the participation of the project team(s) in designing action plans to solve task-related problems of the project.
4. Engaging the project team(s) in the implementation of action plans to solve task-related problems.
5. Engaging the project team(s) in the evaluation of action plans to solve task-related problems.

## Measure items for Teamwork

All the questions were clearly related to the respondent's last project, starting with the words: “In my last project…”

#### Team Communication

1. There was frequent communication within the team.
2. The team members communicated often in spontaneous meetings, phone conversations, etc.
3. The team members communicated mostly directly and personally with each other.
4. There were mediators through whom much communication was conducted (R^[[1]](#footnote-1)^).
5. Project-relevant information was shared openly by all team members.
6. Important information was kept away from other team members in certain situations (R^*^).
7. In our team, there were conflicts regarding the openness of the information flow (R^*^).
8. The team members were happy with the timeliness in which they received information from other team members.
9. The team members were happy with the precision of the information received from other team members.
10. The team members were happy with the usefulness of the information received from other team members.

#### Team Cohesion

1. It was important to the members of our team to be part of this project.
2. The team did not see anything special in this project. (R^*^)^[[2]](#footnote-2)^
3. The team members were strongly attached to this project.
4. The project was important to our team.
5. All members were fully integrated into our team.
6. There were many personal conflicts in our team.(R^*^)
7. There was personal attraction between the members of our team.
8. Our team stuck together.
9. The members of our team felt proud to be part of the team.
10. Every team member felt responsible for maintaining and protecting the team.

#### Team Collaboration

1. We achieved project goals collectively.
2. We had a mutual understanding of the project development process.
3. We informally worked together on project matters.
4. We freely shared ideas, information, and/or resources on project matters.
5. We worked together as a team.

## Measure items for Justice

All the questions were clearly related to the respondent's last project, starting with the words: “In my last project…”

1. Conflicts in the project team were resolved fairly.
2. Team members were recognized when they performed well.
3. Superiors took all suggestions of the team members seriously.
4. The division of responsibilities in the project was fair.

## Measure items for Individual Engagement

1. I really “throw” myself into my work.
2. I devote a lot of effort and energy to my work.
3. I gain considerable pride from performing my job well.
4. I feel passionate and enthusiastic about my job.
5. Performing work is so absorbing that I often forget about the time.
6. I tend to be highly focused when doing my job.

1. R stands for a reverse-scored item [↑](#footnote-ref-1)
2. ^*^ R stands for a reverse-scored item [↑](#footnote-ref-2)
