# Supplementary material for: Shared leadership and project success: The mediational role of individual- and team-related factors
Source: PLoS One. 2026 Feb 13;21(2):e0342268. doi: 10.1371/journal.pone.0342268 (PMC12904471; doi:10.1371/journal.pone.0342268)
Supplement: S2 File — (DOCX) [file pone.0342268.s002.docx]

**S2 File. Measurement instruments used in the study (in Polish)**

## Measure items for Shared Leadership

Wszystkie pytania były związane z ostatnim projektem respondenta, zaczynały się od słów: …”

"W moim ostatnim projekcie

#### Przywództwo transformacyjne (Transformational Leadership)

1. Członkowie mojego zespołu mieli własną wizję, jaka jest rola naszego zespołu w projekcie.

2. Członkowie mojego zespołu kierowali się wyższymi celami lub ideami.

3. Członkowie mojego zespołu rzadko okazywali entuzjazm wobec mojego wysiłku w pracy. R

4. Członkowie mojego zespołu zachęcali mnie do przemyślenia moich pomysłów, mimo że nikt ich wcześniej nie podważał.

5. Członkowie mojego zespołu poszukiwali różnych możliwości rozwiązywania pojawiających się problemów.

6. Członkowie mojego zespołu zachęcali mnie do osiągania więcej niż ode mnie oczekiwano

#### Przywództwo transakcyjne Transactional Leadership

7. Zespołowo dokonywaliśmy ustaleń i trzymaliśmy się ich podczas wspólnej pracy.

8. Kiedy osiągałem dobre wyniki, członkowie mojego zespołu sugerowali, aby podwyższyć moje wynagrodzenie.

9. Członkowie mojego zespołu chwalili mnie, kiedy osiągałem dobre wyniki pracy.

10. Członkowie mojego zespołu byli pełni uznania, kiedy miałem szczególnie dobre wyniki w pracy.

#### Przywództwo partycypacyjne Participative Leadership

11. Wspólnie z zespołem określaliśmy cele w zakresie mojej wydajności pracy.

12. Wspólnie z zespołem decydowaliśmy, jaka powinna być moja docelowa wydajność pracy w projekcie.

13. Wspólnie z zespołem wypracowaliśmy porozumienie co do mojej wydajności.

14. Zespół pomagał mi w opracowaniu celów dotyczących mojej wydajności.

#### Przywództwo wzmacniające dotyczące jednostki (Empowering Leadership - (Individual-related)

15. Członkowie mojego zespołu zachęcali mnie, bym samodzielnie rozwiązywał pojawiające się problemy.

16. Członkowie mojego zespołu namawiali mnie do podejmowania odpowiedzialnych zadań w projekcie.

17. Członkowie mojego zespołu zachęcali mnie do uczenia się nowych rzeczy w projekcie.

18. Członkowie mojego zespołu zachęcali, żebym sobie gratulował, kiedy miałem nowe osiągnięcia.

#### Przywództwo wzmacniające dotyczące zespołu (Empowering Leadership - Team-related)

19. Członkowie mojego zespołu zachęcali mnie do współpracy z innymi członkami.

20. Członkowie mojego zespołu radzili mi, aby łączyć mój wysiłek z wysiłkiem innych osób w zespole.

21. Członkowie mojego zespołu zachęcali mnie do wspólnej pracy.

22. Członkowie mojego zespołu oczekiwali, że nasza współpraca będzie układała się dobrze.

#### Przywództwo awersyjne (Aversive Leadership)

23. Członkowie mojego zespołu grozili mi i zastraszali mnie.

24. Czasem bywałem speszony zachowaniem członków mojego zespołu.

25. Zdarzało się, że członkowie mojego zespołu onieśmielali mnie.

26. Członkowie mojego zespołu wytykali mi, gdy moja praca nie była tak dobra jak praca innych.

## Measure items for Team Building

Wszystkie pytania były wyraźnie związane z ostatnim projektem respondenta, zaczynając od słów: "W moim ostatnim projekcie faktycznie wystąpiły następujące zjawiska:"

#### Ustalanie celów (Goal Setting)

1. Cały zespół uczestniczył w ustalaniu celów projektu.

2. Członkowie zespołu byli zaangażowani w planowanie sposobów realizacji celów projektu.

3. Członkom zespołu wyjaśniono, jakie są podstawowe cele projektu.

4. Zespół w odpowiednim czasie otrzymywał informacje związane z osiąganiem celów projektu*.*

#### Relacje interpersonalne (Interpersonal Relations)

5. Członkowie zespołu byli zachęcani do spotykania się podczas realizacji projektu.

6. Zespół otwarcie rozmawiał o konfliktach.

7. Zespół projektowy szczerze rozmawiał o relacjach między członkami projektu.

8. Zespół projektowy uczestniczył w szkoleniach z zakresu umiejętności komunikacyjnych

9. Zespół projektowy miał możliwość dzielenia się odczuciami w swoim gronie

#### Wyjaśnianie roli (Role Clarification)

10.Każdy członek zespołu miał ustalone role.

11.Członkowie zespołu byli poinformowani o wspólnych obowiązkach.

12.Zespół projektowy był zapoznany z normami projektowymi.

#### Rozwiązywanie problemów (Problem Solving)

13.Zespół angażował się w identyfikację problemów pojawiających się w trakcie realizacji zadań w projekcie.

14.Zespół angażował się w poszukiwanie przyczyn problemów w trakcie realizacji projektu.

15.Zespół projektowy uczestniczył w opracowywaniu sposobów rozwiązywania zaistniałych problemów w projekcie.

16.Zespół angażował się we wdrożenie planu rozwiązywania problemów w realizacji projektu.

17.Zespół dokonywał oceny planu rozwiązywania problemów w projekcie.

## Measure items for Teamwork

Wszystkie pytania były jednoznacznie związane z ostatnim projektem respondenta, zaczynającym się od słów: "W moim ostatnim projekcie..."

#### Komunikacja w zespole (Team Communication)

1. W zespole często komunikowaliśmy się ze sobą.
2. Członkowie zespołu rozmawiali często podczas spontanicznych spotkań, rozmów telefonicznych, itp.
3. Członkowie zespołu porozumiewali się ze sobą głównie bezpośrednio i osobiście.
4. W komunikacji zespołu pomagali mediatorzy. (R^[[1]](#footnote-1)^)
5. Wszyscy członkowie zespołu otwarcie przekazywali dalej informacje na temat projektu.
6. Członkowie zespołu zatrzymywali dla siebie niektóre ważne informacje. (R^*^)
7. W naszym zespole zdarzały się konflikty związane z przepływem informacji. (R^*^)
8. Członkowie zespołu uważali, że informacje otrzymywali od pozostałych członków zespołu w odpowiednim czasie.
9. Członkowie zespołu byli zadowoleni z dokładności informacji otrzymywanych od innych członków zespołu.
10. Członkowie zespołu byli zadowoleni z przydatności informacji otrzymanych od innych członków zespołu.

#### Spójność zespołu (Team Cohesion)

1. Dla członków naszego zespołu udział w projekcie był ważny.
2. Zespół nie widział w tym projekcie niczego szczególnego. (R^*^)^[[2]](#footnote-2)^
3. Członkowie zespołu odczuwali silną więź z tym projektem.
4. Projekt był ważny dla naszego zespołu.
5. W naszym zespole wszyscy członkowie byli w pełni zintegrowani.
6. W ostatnim projekcie było wiele konfliktów między członkami zespołu. (R^*^)
7. Członkowie naszego zespołu darzyli się sympatią.
8. Nasz zespół trzymał się razem.
9. Członkowie naszego zespołu byli dumni, że do niego należą.
10. Każdy członek zespołu czuł się odpowiedzialny za to, by zespół się nie rozpadł i mógł spokojnie pracować

#### Współpraca zespołowa (Team Collaboration)

1. Cele projektu osiągnęliśmy wspólnym wysiłkiem zespołu.
2. W zespole jednakowo rozumieliśmy proces realizacji projektu
3. Pracowaliśmy też nieformalnie nad realizacją projektu.
4. W zespole swobodnie dzieliliśmy się swoimi pomysłami, informacjami i wiedzą dotyczącą projektu.
5. W pracy nad projektem tworzyliśmy zgrany zespół.

## Measure items for Justice

Wszystkie pytania były jednoznacznie związane z ostatnim projektem respondenta, zaczynającym się od słów: "W moim ostatnim projekcie..."

1. Konflikty były rozwiązywane sprawiedliwie.

2. Członkowie zespołu byli doceniani, kiedy dobrze wykonywali swoje zadania.

3. Przełożeni traktowali poważnie wszystkie sugestie członków zespołu.

4. Podział obowiązków był sprawiedliwy.

## Measure items for Individual Engagement

1. Często rzucałem się w wir pracy.

2. Poświęcałem mojej pracy dużo wysiłku i energii.

3. Byłem bardzo dumny, gdy dobrze wykonywałem moją pracę.

4. Czułem pasję i entuzjazm wykonując moją pracę.

5. Praca była tak absorbująca, że często zapominałem o upływającym czasie.

6. W trakcie pracy bywałem bardzo skupiony.

1. R pozycja wg odwróconej skali [↑](#footnote-ref-1)
2. ^*^ pozycja wg odwróconej skali [↑](#footnote-ref-2)
